# Milk Consumption Following Exercise Reduces Subsequent Energy Intake in Female Recreational Exercisers

**DOI:** 10.3390/nu7010293

**Published:** 2015-01-06

**Authors:** Penny Rumbold, Emily Shaw, Lewis James, Emma Stevenson

**Affiliations:** 1 Department of Sport, Exercise and Rehabilitation, Faculty of Health and Life Sciences, Northumbria University, Newcastle upon Tyne, NE1 8ST, UK; E-Mails: emily2.shaw@hotmail.co.uk (E.S.); e.stevenson@northumbria.ac.uk (E.S.); 2 School of Sport, Exercise and Health, Loughborough University, Loughborough, LE11 3TT, UK; E-Mail: L.James@lboro.ac.uk

**Keywords:** females, milk, energy intake, subjective appetite, cycling exercise

## Abstract

The aim of this study was to evaluate the effects of skimmed milk as a recovery drink following moderate–vigorous cycling exercise on subsequent appetite and energy intake in healthy, female recreational exercisers. Utilising a randomised cross-over design, nine female recreational exercisers (19.7 ± 1.3 years) completed a $\dot{\text{V}}{\text{O}}_{2\text{peak}}$ test followed by two main exercise trials. The main trials were conducted following a standardised breakfast. Following 30 min of moderate-vigorous exercise (65% $\dot{\text{V}}{\text{O}}_{2\text{peak}}$), either 600 mL of skimmed milk or 600 mL of orange drink (475 mL orange juice from concentrate, 125 mL water), which were isoenergetic (0.88 MJ), were ingested, followed 60 min later with an *ad libitum* pasta meal. Absolute energy intake was reduced 25.2% ± 16.6% after consuming milk compared to the orange drink (2.39 ± 0.70 *vs*. 3.20 ± 0.84 MJ, respectively; *p* = 0.001). Relative energy intake (in relation to the energy content of the recovery drinks and energy expenditure) was significantly lower after milk consumption compared to the orange drink (1.49 ± 0.72 *vs*. 2.33 ± 0.90 MJ, respectively; *p* = 0.005). There were no differences in AUC (× 1 h) subjective appetite parameters (hunger, fullness and desire to eat) between trials. The consumption of skimmed milk following 30 min of moderate-vigorous cycling exercise reduces subsequent energy intake in female recreational exercisers.

## 1. Introduction

Milk is a nutrient-dense food that contains high quality protein (~34 g/L), carbohydrate (~50 g/L) and electrolytes. Milk contains casein and whey proteins in a ratio of 4:1, which results in slow digestion and absorption of these proteins and, therefore, a sustained elevation of blood amino acid concentrations [1]. These characteristics theoretically make milk an excellent recovery drink to consume following both resistance and endurance exercise. Following exercise, the carbohydrates in milk provide a substrate for muscle glycogen [2], whilst the protein stimulates muscle protein synthesis [3]. Studies have reported a greater restoration of exercise capacity following the ingestion of a milk-based recovery drink compared to other commercially-available recovery drinks [4,5]. Work from our laboratory has shown that ingestion of milk post-exercise can alleviate the negative effects of exercise-induced muscle damage [6,7], and combining milk intake with resistance exercise training has been shown to enhance gains in muscle mass in young men [3,8] and women [9].

Milk has also been shown to be more satiating [10,11] compared to carbohydrate drinks, an effect probably attributable to the protein contained in milk [12,13]. Dove* et al.* [11] showed that consumption of milk at breakfast increased perceptions of satiety and attenuated energy intake at lunch 4 h later compared to an isocaloric fruit drink in overweight men and women. Similarly, Harper* et al.* [10] reported that satiety was increased following ingestion of a chocolate milk drink compared to an isoenergetic carbonated drink, but this did not translate into a reduced energy intake at a meal 30 min later.

Previous research has demonstrated differences in appetite regulation between males and females in response to alterations in energy balance [14]. Researchers have highlighted observed sex differences pertaining to the effect of exercise on appetite-regulating hormones [14]. The cited authors identified that in women, regardless of energy status, exercise stimulated orexigenic-regulating hormones. This suggests that in women, energy conservation might be promoted during exercise training via alterations in appetite-regulating hormones, which might stimulate energy intake in spite of energy status. This supports the notion that compensatory energy intake in females tends to be accentuated and more powerful than in males following energy deficit [14].

It appears that milk is an excellent post-exercise recovery drink, which, due to its appetite-suppressing potential, might help enhance recovery from exercise whilst also helping to promote a negative energy balance. Many individuals, particularly women, exercise on a regular basis for weight loss or weight maintenance purposes [15], and therefore, milk could be an ideal post-exercise drink to consume. 

Therefore, the aim of this study was to investigate the effect of consuming milk as a recovery drink following moderate intensity exercise on subsequent appetite and energy intake in recreationally-active females.

## 2. Experimental Section 

### 2.1. Design

A within-subjects, randomised, cross-over design was used to explore the effects of skimmed milk or orange drink consumption on subsequent appetite and energy intake, following 30 min of cycling exercise, in female recreational exercisers.

The study was approved by the Faculty of Health and Life Sciences Research Ethics Committee at the University of Northumbria. Written informed consent was obtained from participants prior to data collection. Participants were notified that the study was to investigate general aspects of diet and, thus, were not informed about the specific nature of the study to ensure no alterations to eating behaviour.

### 2.2. Participants

Nine female recreational exercisers (mean ± SD), aged 19.7 ± 1.3 years, mass 61.7 ± 4.4 kg, stature 167.0 ± 5.3 cm, BMI 22.1 ± 1.7 kg/m^2^, $\dot{\text{V}}{\text{O}}_{2\text{peak}}$ 45.7 ± 13.4 mL/kg/min, were recruited to take part in the study. Five participants were oral contraceptive users. Participants were classified as normal weight (BMI 19–25 kg/m^2^) and were non-restrained (<7) eaters, according to the Three Factor Eating Questionnaire [16]. In addition, a menstrual cycle question was also completed to ensure that the main trials were conducted in the early follicular phase (Days 1–14).

### 2.3. Preliminary Measures

Participants carried out a discontinuous $\dot{\text{V}}{\text{O}}_{2\text{peak}}$ protocol, on a cycle ergometer (Monark Weight Ergometer 839 E, Varberg, Sweden), completing an initial stage at 70 W, and from there, the work load was increased by 35 W, until volitional fatigue. The increment of the subsequent stage was determined from the heart rate and Ratings of Perceived Exertion (RPE) values given in previous stages. Expired air was collected using 200-L Douglas bags (Plysu Industrial Ltd Milton Keynes) during the final minute of each stage, and the heart rate (bpm) (Polar Heart Rate Monitor, Polar O.Y. Finland) and RPE were taken in the final 15 s of each stage. Expired air was examined using calibrated gas analysis equipment (Servomex combined O_2_ and CO_2_ Analyser, Servomex Ltd., Crowborough). From this, a work rate of 65% $\dot{\text{V}}{\text{O}}_{2\text{peak}}$ was established for each individual using a regression for the linear function of oxygen uptake at each of the work rates. Maximal oxygen uptake was measured against the work rate (W) of each of the steady-state work rates and maximal power output to derive a value for the subsequent exercise protocol testing days [17]. 

### 2.4. Protocol

Participants were asked to attend the laboratory on three separate occasions (preliminary measures, followed by two main trials). All main trials were carried out during the follicular phase of the menstrual cycle (Days 1–14; on average, there were 10 ± 3 days between trials). Participants were asked to refrain from caffeine, alcohol and vigorous physical activity 12 h prior to all main trials and arrive at the laboratory following a 10-h overnight fast. As per our previous studies [18,19], participants were asked to replicate their food and fluid intake and portion sizes for their evening meal prior to each trial, using photocopies of the first week’s self-reported, weighed food diaries.

**Figure 1:**
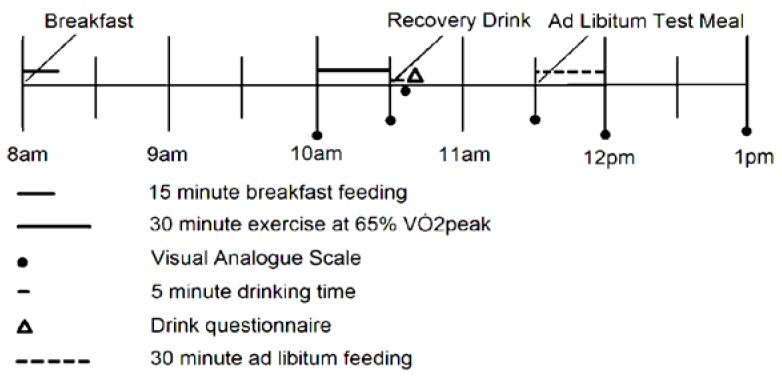
Protocol outline.

The participants were asked to consume a pre-prepared breakfast at 08:00 at home and subsequently arrive at the laboratory at 09:45. The 30-min continuous exercise was conducted on the cycle ergometer at 65% ± 4% of the participants individual $\dot{\text{V}}{\text{O}}_{2\text{peak}}$. Expired air (L·min^−1^), heart rate (bpm) and RPE scores were obtained at the following time points: 9–10 min, 19–20 min and 29–30 min. The recovery drink of either skimmed milk or an orange drink was provided immediately after exercise completion, in an opaque bottle, and consumed within 5 min. An *ad libitum* pasta meal was made available to the participants 60 min after drink ingestion with a maximum feeding time of 30 min. Subjective appetite sensations (hunger, prospective food consumption and fullness) were assessed using visual analogue scales (VAS) at the time points identified in Figure 1.

### 2.5. Fixed Energy Breakfast

The daily energy requirement (DER) was calculated by multiplying basal metabolic rate (predicted using the Schofield equation) by a physical activity level of 1.7. This enabled the amount of rice snaps and semi-skimmed milk (Tesco, UK) to be calculated for each individual in order to meet 10% of their DER. The standardised fixed-energy breakfast meal consisted of a cereal:milk ratio of 30 g:120 mL and delivered fat, protein and carbohydrate with a macronutrient composition of 14, 14 and 72%, respectively [12].

### 2.6. Test Beverages

The two recovery drinks provided to the participants were 600 mL of skimmed milk (Tesco, UK) and 600 mL of an orange drink, consisting of 475 mL orange juice from concentrate (Tesco, UK) and 125 mL water to balance the energy content of the drinks (Table 1). All drinks were kept refrigerated (~5 °C) until they were served to the participants.

**Table 1:** Composition of the recovery drinks.

	Skimmed Milk ^1^ (600 mL)	Fruit Juice ^2^ (475 mL orange, 125 mL water)
Energy		
Per portion (MJ)	0.88	0.88
Protein		
Per portion (g)	20.4	2.4
Fat		
Per portion (g)	0.6	0
Carbohydrate		
Per portion (g)	30.0	49.8
Calcium Per portion (mg)	744	0
Glycaemic index	30	46–53

^1^ Skimmed milk, Tesco, UK. ^2^ Orange juice smooth from concentrate, Tesco, UK.

### 2.7. Ad libitum Test Meal

The *ad libitum* test meal consisted of fusilli pasta (Tesco, UK), bolognaise sauce (Tesco, UK), cheddar cheese (British Mature, Tesco, UK) and olive oil (“Drizzle”, Tesco, UK), with preparation and cooking protocols standardised and repeated across all main trials. The meal provided fat, protein and carbohydrate in the following proportions: 34%, 14% and 52%, respectively. Participants were provided with a quiet environment in which to consume the *ad libitum* pasta meal and were instructed to eat until they felt comfortably full. Participants were provided with a full bowl of pasta, which was continuously topped up by the research team throughout consumption. This meant that participants could not simply finish one bowl of pasta, which may have been perceived as a standard portion. The amount of food consumed was calculated and recorded using electronic measuring scales (Sartorius TE6100, A.G Germany), by subtracting waste from the total pre-eating weight administered. We have successfully used this meal to assess energy intake in other energy regulation studies [12,19,20].

### 2.8. Subjective Sensations

Subjective appetite responses were rated on 100-mm horizontal lines, to each of the following questions: “How hungry do you feel now?” anchored by very hungry (100) and not at all hungry (0); prospective food consumption and “How much would you like to eat now?” anchored by a lot (100) and nothing at all (0); “How full do you feel now?” anchored by very full (100) and not full at all (0). All VAS were measured by hand by one researcher, from the minimum score of 0 mm to the maximum score of 100 mm.

### 2.9. Data Analysis

Excel (Version: 2010) was used for all statistical analyses. The means ± SD were calculated for all data with the exception of VAS data, where the means ± SE were used. VAS ratings for subjective appetite sensations (hunger, prospective food consumption and fullness) were calculated as time-averaged area under the curve (AUC) for the post drink, pre-meal periods (1 h). Absolute energy intake (MJ) was considered as the absolute amount of pasta consumed at the test meal. Relative energy intake at the pasta test meal (in relation to the energy content of the recovery drinks and energy expenditure during the cycling exercise) (MJ) was calculated by totalling absolute energy intake at the pasta meal with the energy content of the drinks and subtracting the energy expended during the cycling exercise. Subsequently, exercise-induced energy expenditure (MJ), absolute and relative energy intake (MJ), as well as VAS appetite (hunger, prospective food consumption and fullness: AUC × 1 h) for the milk and orange drink trials were analysed using paired samples *t*-tests. Statistical significance was accepted at *p* < 0.05 for all analyses.

## 3. Results

### 3.1. Energy Expenditure

As intended, the energy cost of the 30-min cycling exercise at 65% $\dot{\text{V}}{\text{O}}_{2\text{peak}}$ was not different between trials, when the milk or orange was provided as a recovery drink (1.78 ± 0.02 *vs*. 1.74 ± 0.04 MJ, respectively; *p* = 0.382).

### 3.2. Energy Intake

**Figure 2:**
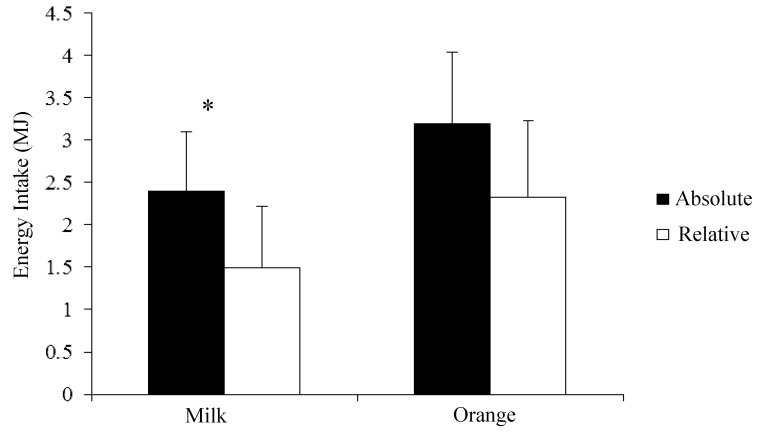
Mean (± SD) absolute and relative energy intake (MJ) (*ad libitum* test meal), following 30 min of moderate–vigorous exercise at 65% $\dot{\text{V}}{\text{O}}_{2\text{peak}}$ between milk and orange juice trials (*n* = 9). * Energy intake significantly lower for absolute and relative conditions (*p* = 0.001 and *p* = 0.005, respectively).

Absolute energy intake at the pasta test meal was significantly less following consumption of milk compared to the orange drink (2.39 ± 0.70 *vs*. 3.20 ± 0.84 MJ, respectively; *p* = 0.001) with a mean reduction of 0.81 ± 0.50 MJ (25.2% ± 14.1%). Relative energy intake at the pasta test meal (in relation to the energy content of the recovery drinks and energy expenditure during the cycling exercise) was also significantly lower after milk consumption compared to the orange drink (1.49 ± 0.72 *vs*. 2.33 ± 0.90 MJ, respectively; *p* = 0.005) with a mean reduction of 0.83 ± 0.54 MJ (37.4% ± 27.2%) (Figure 2).

### 3.3. Subjective Appetite Sensations

**Figure 3:**
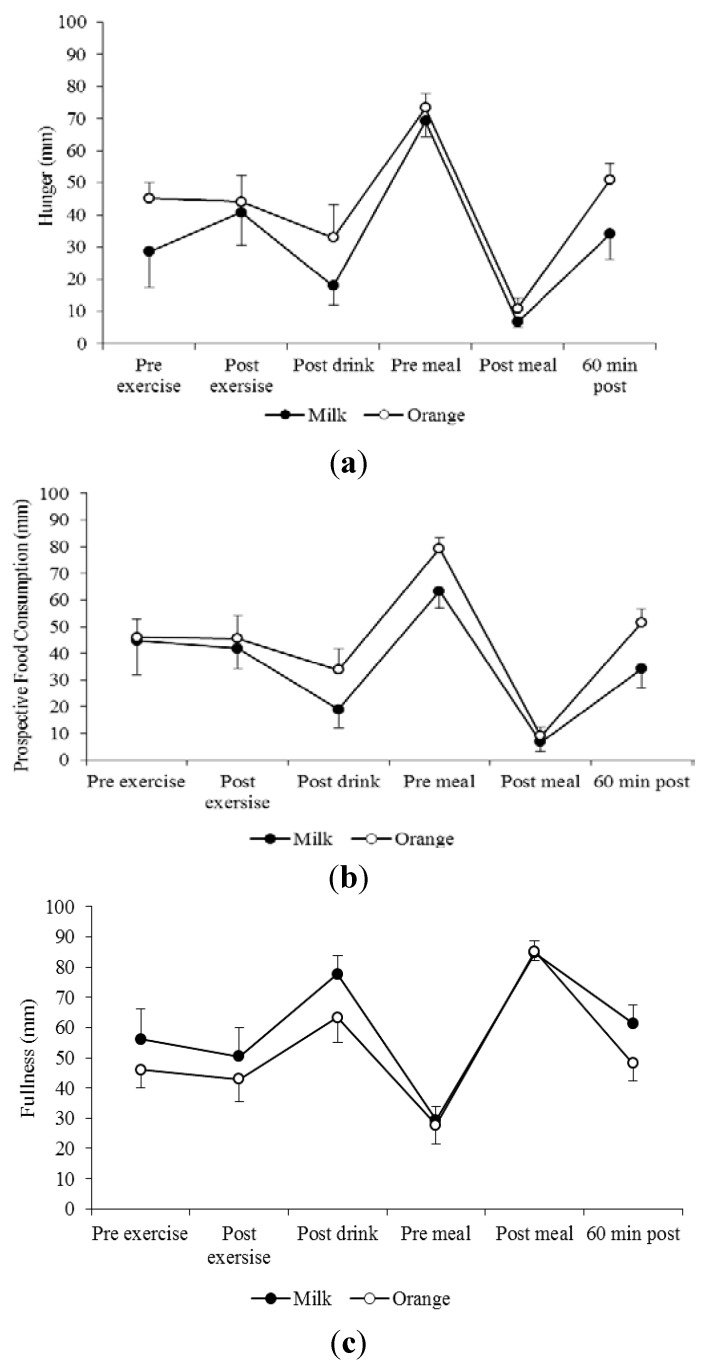
Subjective appetite (mean ± SE (**a**) hunger (mm); (**b**) prospective food consumption (mm); and (**c**) fullness (mm)) ratings on the visual analogue scales for the milk (●) and orange juice (○) trials (*n* = 9).

There were no observed differences in AUC (× 1 h) subjective appetite between trials for hunger (*p* = 0.267), prospective food consumption (*p* = 0.063) and fullness (*p* = 0.410) (Figure 3a–c).

## 4. Discussion

Taking a novel approach, the present study was the first to investigate the effects of skimmed milk and an orange drink on subsequent *ad libitum* energy intake following moderate intensity cycling exercise in recreational females. Although other studies using dietary interventions have highlighted the satiating effect of milk on subsequent energy intake in both males and females [11,21], none have investigated this effect succeeding the completion of exercise. This study therefore identified that the consumption of skimmed milk following 30 min of moderate–vigorous cycling exercise resulted in a significant reduction in subsequent energy intake in female recreational exercisers (0.81 MJ or ~25% lower compared to the orange juice trial). Such an alteration in eating behaviour was not accompanied by any fluctuations in subjective appetite (hunger, prospective food consumption or fullness). Despite this, the reduction in energy intake of 0.81 MJ can be described as a worthwhile change, given that several authors have identified energy intake deficits of 170 kcal (712 kJ) and 175 ± 45 kcal (733 ± 188 kJ) per day [22,23] to be “clinically” meaningful from a weight loss/maintenance perspective. 

Indeed, there are several plausible explanations for the positive observations with regards to the reduction in energy intake following the consumption of skimmed milk in the present study. As demonstrated in Table 1, milk is a significant source of dietary protein, which has been found to exert greater satiating effects when paralleled with carbohydrate-rich drinks, such as fruit juice, which do not contain protein [2,10,11,24]. In addition, several studies have reported a significantly lower energy intake and increased subjective satiety following a high-protein meal [12], with appetite suppression being superior when compared to isoenergetic high-carbohydrate and high-fat meals [13]. The mechanism behind this effect has been suggested to be related to elevated levels of cholecystokinin (CCK) and glucagon-like peptide-1 (GLP)-1, accompanied by reductions in circulating concentrations of ghrelin [25,26]. Indeed, recent studies have proposed the satiating effects of bioactive foods, such as milk and dairy products, to be modulated by various anorexic peptides, namely CCK and PYY_3-36_ [27,28]. In addition to the protein content, the type of protein in dairy products may influence satiety (see Bendtsen* et al.* [29] for a review of the effects of dairy protein on appetite). The composition of milk protein is ~80% casein and ~20% whey by mass [3]. The metabolic response to protein differs by the type consumed, with whey consumption stimulating muscle protein synthesis to a greater extent than casein and soy [30,31]. This is likely to be due to differences in the amino acid composition (in particular, the greater leucine content of whey) and the faster rate of intestinal absorption [31,32]. These properties of protein sources that produce differential muscle protein synthesis rates are likely to manifest in changes in body composition over time, but whether they also affect the appetite response to consumption is not yet clear.

Furthermore, it has been postulated that the type of carbohydrate, lipid composition and the calcium content of milk may also contribute to the beneficial effects towards appetite control. The primary source of carbohydrate in milk is the disaccharide, lactose, comprised of glucose and galactose linked by a glycosidic bond, which is hydrolysed by lactase. Lactose is not as sweet as glucose and has a glycaemic index (GI) of 43 [33], regarded as low GI. Although the impact of the GI of foods *per se* is not clear, lactose does appear to be a particularly satiating sugar. When males were fed 1025 kJ preloads containing 56 g, of either glucose or lactose, energy intake at a subsequent meal (180 min post-preload) was 11% lower (*p* < 0.05) with lactose* vs.* glucose [26]. Appetite sensations were also suppressed, as were circulating ghrelin concentrations. Compared to glucose, galactose results in lower postprandial glycaemia [15] and suppresses hunger sensations [34]. Thus, it is plausible that lactose and galactose contribute to satiety following the ingestion of dairy products. 

Milk lipids are comprised of ~95% triacylglycerol, ~2% diacylglycerol, ~1% phospholipids and cholesterol and non-esterified fatty acids contributing <0.5% each [35]. Approximately 64% of the lipid content of milk is from saturated fatty acids, with a substantial contribution (~26%) from monounsaturated fatty acids and smaller contributions from polyunsaturated fatty acids and trans-fatty acids (both ~3%). When matched for energy, fat is generally less satiating than protein and carbohydrate. This is purely an effect of energy density rather than fat *per se* [36]. Fat can also influence food texture, which may suppress appetite due to increased viscosity or potentially enhance appetite through an increase in palatability (particularly when combined with sweet flavours [37]). The structure of the fat globules in milk are worthy of consideration, influencing metabolism and appetite. Emulsification of milk fat (serving to increase the surface area by ~70,000-fold) potentiates postprandial circulating apolipoprotein B-48 concentrations and exogenous lipid oxidation. Interestingly, obese participants in the study by Vors* et al.* [38] reported significantly greater hunger ratings following consumption of the emulsified milk fat, compared to non-emulsified milk fat. There was no significant difference in appetite ratings between trials in non-obese participants, although the trend was similar. Accordingly, the structure of dairy fat is likely to influence appetite, at least in certain populations, potentially through an impact on the rate of digestion and absorption.

Finally, the calcium content of meals has also been linked to postprandial circulating glucose-dependent insulinotropic peptide (GIP)_1-42_, GLP-1 and insulin concentrations [39], which are known to suppress appetite [40,41]. Recent evidence has implied that calcium influences appetite, both acutely and chronically. After consumption of a high-calcium and vitamin D meal, subsequent 24-h energy intake is reduced [42]. A mechanism to explain this effect could be the stimulation of the calcium sensing receptor (CaSR), which is ubiquitously expressed in the human gastrointestinal tract [43]. In a model using an isolated rodent intestine, the presence of calcium potentiates the response of K- and L-cells to l-amino acids to secrete GIP, GLP-1 and peptide tyrosine tyrosine (PYY), an effect that is abolished with a CaSR antagonist and potentiated with a CaSR agonist [44]. Based on* in vitro* data, CaSR-stimulated peptide secretion seems to act through both cAMP and intracellular calcium signalling pathways [39]. In humans, calcium co-ingestion with a meal enhances the postprandial circulating concentrations of GIP_1-42_ and total GLP-1 [39]. Given the well-known role of GIP and GLP-1 in insulin secretion, it is not surprising that postprandial insulinaemia is also transiently elevated [39,45]. As insulin and GLP-1 both suppress appetite upon administration [40,41], this provides a mechanism by which calcium influences postprandial satiety.

Given the intention of conducting a laboratory-based study with elements of high ecological validity (the provision of two representative recovery drinks), we believe various aspects of the study methodology to have been extremely robust (isoenergetic recovery drinks; rigorous assessment of subjective appetite and energy intake). A potential limitation of the present study was that we did not include a water control trial; however, our intention was to explore the potential satiating effects of drink products which have been shown to be beneficial for recreational females to consume to promote recovery following exercise (*i.e.*, carbohydrate- and milk-based drinks) [9]. Furthermore, we were unable to blind the drinks from the participants, as doing so would have modified the characteristics of the drinks, which was not desired. Finally, no hormonal indicators of appetite were sought. Therefore, there is a requirement for both subjective appetite and short-term hormonal regulators of appetite to be assessed concurrently in future dairy-focused appetite control studies. Findings from the present study provide a strong rationale for this work to be advanced by exploring objective measures of appetite, such as GIP_1-42_ and active GLP-1, to establish the mechanistic link between milk consumption post exercise and appetite regulation.

## 5. Conclusions

In conclusion, the present study demonstrated that the consumption of skimmed milk following 30 min of moderate-vigorous cycling exercise resulted in a significant reduction in acute energy intake in female recreational exercisers.

## References

[1] Bos C., Metges C.C., Gaudichon C., Petzke K.J., Pueyo M.E., Morens C., Everwand J., Benamouzig R., Tome D. (2003). Postprandial kinetics of dietary amino acids are the main determinant of their metabolism after soy or milk protein ingestion in humans. J. Nutr..

[2] Ferguson-Stegall L., McCleave E., Ding Z., Doerner Iii P.G., Liu Y., Wang B., Healy M., Kleinert M., Dessard B., Lassiter D.G. (2011). Aerobic exercise training adaptations are increased by postexercise carbohydrate-protein supplementation. J. Nutr. Metab..

[3] Wilkinson S.B., Tarnopolsky M.A., Macdonald M.J., Macdonald J.R., Armstrong D., Phillips S.M. (2007). Consumption of fluid skim milk promotes greater muscle protein accretion after resistance exercise than does consumption of an isonitrogenous and isoenergetic soy-protein beverage. Am. J. Clin. Nutr..

[4] Karp J.R., Johnston J.D., Tecklenburg S., Mickleborough T.D., Fly A.D., Stager J.M. (2006). Chocolate milk as a post-exercise recovery aid. Int. J. Sport Nutr. Exerc. Metab..

[5] Thomas K., Morris P., Stevenson E. (2009). Improved endurance capacity following chocolate milk consumption compared with 2 commercially available sport drinks. Appl. Physiol. Nutr. Metab..

[6] Cockburn E., Hayes P.R., French D.N., Stevenson E., St Clair Gibson A. (2008). Acute milk-based protein-cho supplementation attenuates exercise-induced muscle damage. Appl. Physiol. Nutr. Metab..

[7] Cockburn E., Stevenson E., Hayes P.R., Robson-Ansley P., Howatson G. (2010). Effect of milk-based carbohydrate-protein supplement timing on the attenuation of exercise-induced muscle damage. Appl. Physiol. Nutr. Metab..

[8] Hartman J.W., Tang J.E., Wilkinson S.B., Tarnopolsky M.A., Lawrence R.L., Fullerton A.V., Phillips S.M. (2007). Consumption of fat-free fluid milk after resistance exercise promotes greater lean mass accretion than does consumption of soy or carbohydrate in young, novice, male weightlifters. Am. J. Clin. Nutr..

[9] Josse A.R., Tang J.E., Tarnopolsky M.A., Phillips S.M. (2010). Body composition and strength changes in women with milk and resistance exercise. Med. Sci. Sports Exerc..

[10] Harper A., James A., Flint A., Astrup A. (2007). Increased satiety after intake of a chocolate milk drink compared with a carbonated beverage, but no difference in subsequent *ad libitum* lunch intake. Br. J. Nutr..

[11] Dove E.R., Hodgson J.M., Puddey I.B., Beilin L.J., Lee Y.P., Mori T.A. (2009). Skim milk compared with a fruit drink acutely reduces appetite and energy intake in overweight men and women. Am. J. Clin. Nutr..

[12] Astbury N.M., Stevenson E.J., Morris P., Taylor M.A., Macdonald I.A. (2010). Dose-response effect of a whey protein preload on within-day energy intake in lean subjects. Br. J. Nutr..

[13] Stubbs R.J., van Wyk M.C., Johnstone A.M., Harbron C.G. (1996). Breakfasts high in protein, fat or carbohydrate: Effect on within-day appetite and energy balance. Eur. J. Clin. Nutr..

[14] Hagobian T.A., Sharoff C.G., Stephens B.R., Wade G.N., Silva J.E., Chipkin S.R., Braun B. (2009). Effects of exercise on energy-regulating hormones and appetite in men and women. Am. J. Physiol. Regul. Integr. Comp. Physiol..

[15] Duckworth L.C., Backhouse S.H., Stevenson E.J. (2013). The effect of galactose ingestion on affect and perceived exertion in recreationally active females. Appetite.

[16] Stunkard A.J., Messick S. (1985). The three factor eating questionnaire to measure dietary restraint, disinhibition and hunger. J. Psychosom. Res..

[17] Stevenson E.J., Astbury N.M., Simpson E.J., Taylor M.A., Macdonald I.A. (2009). Fat oxidation during exercise and satiety during recovery are increased following a low-glycemic index breakfast in sedentary women. J. Nutr..

[18] Rumbold P.L.S., Gibson A.S.C., Allsop S., Stevenson E., Dodd-Reynolds C.J. (2011). Energy intake and appetite following netball exercise over 5 days in trained 13–15 year old girls. Appetite.

[19] Rumbold P.L.S., Gibson A.S., Stevenson E.J., King J.A., Stensel D.J., Dodd-Reynolds C.J. (2013). Influence of netball-based exercise on energy intake, subjective appetite and plasma acylated ghrelin in adolescent girls. Appl. Physiol. Nutr. Metab..

[20] Gonzalez J.T., Veasey R.C., Rumbold P.L., Stevenson E.J. (2013). Breakfast and exercise contingently affect postprandial metabolism and energy balance in physically active males. Br. J. Nutr..

[21] Dougkas A., Minihane A.M., Givens D.I., Reynolds C.K., Yaqoob P. (2012). Differential effects of dairy snacks on appetite, but not overall energy intake. Br. J. Nutr..

[22] Hill J.O., Wyatt H.R., Reed G.W., Peters J.C. (2003). Obesity and the environment: Where do we go from here?. Science.

[23] Wynne K., Stanley S., McGowan B., Bloom S. (2005). Appetite control. J. Endocrinol..

[24] Maersk M., Belza A., Holst J.J., Fenger-Gron M., Pedersen S.B., Astrup A., Richelsen B. (2012). Satiety scores and satiety hormone response after sucrose-sweetened soft drink compared with isocaloric semi-skimmed milk and with non-caloric soft drink: A controlled trial. Eur. J. Clin. Nutr..

[25] Bowen J., Noakes M., Trenerry C., Clifton P.M. (2006). Energy intake, ghrelin, and cholecystokinin after different carbohydrate and protein preloads in overweight men. J. Clin. Endocrinol. Metab..

[26] Bowen J., Noakes M., Clifton P.M. (2006). Appetite regulatory hormone responses to various dietary proteins differ by body mass index status despite similar reductions in ad libitum energy intake. J. Clin. Endocrinol. Metab..

[27] Soenen S., Westerterp-Plantenga M.S. (2007). No differences in satiety or energy intake after high-fructose corn syrup, sucrose, or milk preloads. Am. J. Clin. Nutr..

[28] Diepvens K., Haberer D., Westerterp-Plantenga M. (2007). Different proteins and biopeptides differently affect satiety and anorexigenic/orexigenic hormones in healthy humans. Int. J. Obes..

[29] Bendtsen LQ., Lorenzen J.K., Bendsen N.T., Rasmussen C., Astrup A. (2013). Effect of dairy proteins on appetite, energy expenditure, body weight, and composition: A review of the evidence from controlled clinical trials. Adv. Nutr..

[30] Tang J.E., Moore D.R., Kujbida G.W., Tarnopolsky M.A., Phillips S.M. (2009). Ingestion of whey hydrolysate, casein, or soy protein isolate: Effects on mixed muscle protein synthesis at rest and following resistance exercise in young men. J. Appl. Physiol..

[31] Pennings B., Boirie Y., Senden J.M., Gijsen A.P., Kuipers H., van Loon L.J. (2011). Whey protein stimulates postprandial muscle protein accretion more effectively than do casein and casein hydrolysate in older men. Am. J. Clin. Nutr..

[32] Wall B.T., Hamer H.M., de Lange A., Kiskini A., Groen B.B., Senden J.M., Gijsen A.P., Verdijk L.B., van Loon L.J. (2013). Leucine co-ingestion improves post-prandial muscle protein accretion in elderly men. Clin. Nutr..

[33] Gannon M.C., Nuttall F.Q., Krezowski P.A., Billington C.J., Parker S. (1986). The serum insulin and plasma glucose responses to milk and fruit products in type 2 (non-insulin-dependent) diabetic patients. Diabetologia.

[34] Duckworth L.C., Backhouse S.H., Stevenson E.J., Ohara J.P. (2013). Effect of galactose ingestion before and during exercise on substrate oxidation and subsequent energy intake in females. Int. J. Sport Nutr. Exerc. Metab..

[35] Haug A., Hostmark A.T., Harstad O.M. (2007). Bovine milk in human nutrition—A review. Lipids Health Dis..

[36] Bell E.A., Rolls B.J. (2001). Energy density of foods affects energy intake across multiple levels of fat content in lean and obese women. Am. J. Clin. Nutr..

[37] Green S.M., Blundell J.E. (1996). Effect of fat- and sucrose-containing foods on the size of eating episodes and energy intake in lean dietary restrained and unrestrained females: Potential for causing overconsumption. Eur. J. Clin. Nutr..

[38] Vors C., Pineau G., Gabert L., Drai J., Louche-Pélissier C., Defoort C., Lairon D., Désage M., Danthine S., Lambert-Porcheron S. (2013). Modulating absorption and postprandial handling of dietary fatty acids by structuring fat in the meal: A randomized crossover clinical trial. Am. J. Clin. Nutr..

[39] Gonzalez J.T., Stevenson E.J. (2014). Calcium co-ingestion augments postprandial glucose-dependent insulinotropic peptide1–42, glucagon-like peptide-1 and insulin concentrations in humans. Eur. J. Nutr..

[40] Air E.L., Benoit S.C., Blake Smith K.A., Clegg D.J., Woods S.C. (2002). Acute third ventricular administration of insulin decreases food intake in two paradigms. Pharmacol. Biochem. Behav..

[41] Verdich C., Flint A., Gutzwiller J.P., Naslund E., Beglinger C., Hellstrom P.M., Long S.J., Morgan L.M., Holst J.J., Astrup A. (2001). A meta-analysis of the effect of glucagon-like peptide-1 (7–36) amide on ad libitum energy intake in humans. J. Clin. Endocrinol. Metab..

[42] Ping-Delfos W.C., Soares M. (2011). Diet induced thermogenesis, fat oxidation and food intake following sequential meals: Influence of calcium and vitamin D. Clin. Nutr..

[43] Geibel J.P., Hebert S.C. (2009). The functions and roles of the extracellular Ca^2+^-sensing receptor along the gastrointestinal tract. Ann. Rev. Phys..

[44] Mace O.J., Schindler M., Patel S. (2012). The regulation of K- and L-cell activity by GLUT2 and the calcium-sensing receptor CasR in rat small intestine. J. Physiol..

[45] Gonzalez J.T., Rumbold P.L.S., Stevenson E.J. (2013). Appetite sensations at rest, during exercise and recovery: Impact of a high-calcium meal. Appl. Physiol. Nutr. Metab..

